# Disease Course and Long-Term Outcomes in Adult IgA Vasculitis Nephritis: A Prospective Observational Study

**DOI:** 10.3390/diagnostics15080957

**Published:** 2025-04-10

**Authors:** Fatih Yıldırım, Muhammet Emin Kutu, Yalkın Çalık, Kübra Kalkan, Gamze Akkuzu, Duygu Sevinç Özgür, Bilgin Karaalioğlu, Rabia Deniz, Gül Güzelant Özköse, Burak İnce, Cemal Bes

**Affiliations:** 1 Department of Rheumatology, Başakşehir Çam and Sakura City Hospital, University of Health Sciences, 34480 İstanbul, Turkey; kalkankubra86@gmail.com (K.K.); gamzeakkuzu89@hotmail.com (G.A.); duygu_se_a@hotmail.com (D.S.Ö.); bilginkaraalioglu@gmail.com (B.K.); guzelant_gul@hotmail.com (G.G.Ö.); drburakince@gmail.com (B.İ.); cemalbes@hotmail.com (C.B.); 2 Department of Rheumatology, Bakırköy Dr Sadi Konuk Training and Research Hospital, University of Health Sciences, 34140 İstanbul, Turkey; mekutu@hotmail.com (M.E.K.); yclk04@hotmail.com (Y.Ç.)

**Keywords:** IgA vasculitis, nephritis, prognosis, mortality, ESRD

## Abstract

**Background/Objectives**: A limited number of previous studies have reported high rates of end-stage renal disease (ESRD) in adults with IgA vasculitis nephritis (IgAVN). Despite the high prevalence of the disease and the high rates of ESRD reported in the literature, no specific guidelines for adult patients have been established and there is no consensus on the management of the disease. This study aimed to prospectively investigate adults with IgAVN from a broad perspective. **Methods**: This investigation was designed as a prospective observational study and was conducted between 01.02.2022 and 01.10.2024. A total of 49 newly diagnosed adult (>18 years) patients with IgAVN were regularly followed up. At the end of the study, the renal remission rates, factors influencing remission, treatment data, treatment-related adverse events, and disease outcomes were determined. **Results**: The median follow-up time was 22 (IQR: 11–24) months. A total of 42 patients (87%) received immunosuppressive treatment in addition to the initial glucocorticoid treatment. Azathioprine (AZA) was the preferred (41%) first steroid-sparing agent. ESRD occurred in only one patient (2%), while a total of ten patients (20%) had an unfavorable outcome. The rate of nephrotic-range proteinuria (NRP) was significantly higher in the patients who did not achieve renal remission at the end of the 12-month follow-up period (9,7% vs. 60%; *p* = 0.02) and NRP was an independent risk factor for unfavorable outcomes [OR: 17.18; 95% CI: 1.31–224.95; *p* = 0.03]. A total of 16% of the patients developed an infection that required hospitalization during follow-up; these patients had a higher rate of IgAVN-associated acute kidney injury (62.5% vs. 22%; *p* = 0.02) and were significantly older (mean: 46 ± 15.3 vs. 65 ± 13.3; *p* = 0.002). One patient died of sepsis at 4 months and another died of a myocardial infarction at 32 months. **Conclusions**: These results suggest that adults with IgVAN do not have a high rate of ESRD if they receive effective immunosuppressive therapy. However, immunosuppressive therapy is associated with an increased risk of infection, particularly in the elderly. The presence of NRP is associated with lower long-term remission rates and has a predictive value for unfavorable outcomes.

## 1. Introduction

Immunoglobulin A vasculitis (IgAV), also known as Henoch–Schönlein purpura (HSP), is a type of primary vasculitis characterized by the deposition of IgA1-immune complexes in small vessels [1]. Its annual incidence in adults has been reported to vary from 0.3/100,000 to 5.1/100,000 [2,3,4]. The disease classically involves the skin, joints, gastrointestinal tract, and kidneys. Renal involvement is the most common cause of morbidity and mortality and is the main determinant of the prognosis in patients with IgAV [5,6]. The renal involvement of the disease is essentially an immune complex glomerulonephritis and is termed IgA vasculitis nephritis (IgAVN) [7]. The rate of IgAVN in adult patients with IgAV has been reported to vary between 45% and 85% [8]. Hematuria is the most common and first manifestation of IgAVN, but proteinuria, nephrotic syndrome, nephritic syndrome, and acute kidney injury may also occur due to the disease [9]. Most of the data in the literature on IgAVN are derived from studies conducted in children. Studies comparing pediatric and adult patients with IgAVN have shown that the disease has a worse prognosis in adults [10,11]. The aim of this study was to prospectively investigate the disease course, outcomes, and treatment options in adults with IgAVN.

## 2. Materials and Methods

### 2.1. Study Design and Population

This investigation was designed as a prospective observational study and was conducted between February 2022 and October 2024 at the University of Health Sciences Başakşehir Çam and Sakura City Hospital Department of Rheumatology. A total of 49 patients aged ≥18 years with histopathological or clinically diagnosed IgAVN were included in the study. Patients with isolated IgA nephropathy and patients whose renal findings could be explained by another concurrent disease were excluded. All the patients were re-examined at 3-month intervals. Their renal functions, urine analysis results, proteinuria level, medications and doses, adverse events, and vasculitis damage scores were recorded at each visit. At the end of the study, the long-term (12-month) renal outcomes of the patients and the factors influencing these outcomes were determined. This study was approved by the hospital ethics committee (approval number: KAEK/2022.11.361) and was conducted according to the guidelines of the Declaration of Helsinki. Informed consent was obtained from all the patients.

### 2.2. Clinical Definitions

The EULAR/PRINTO/PRES classification criteria were used for the diagnosis of IgAV [12]. Renal involvement was defined as the presence of proteinuria (≥150 mg/24 h or ≥150 mg/g spot urine protein/creatinine ratio) and/or hematuria (≥5 red cells per high-power field). Acute kidney injury (AKI) was defined as an increase in serum creatinine of 0.3 mg/dL within 48 h or an increase in baseline serum creatinine of ≥50% within 7 days. The definitions of renal conditions (proteinuria, hematuria, AKI, CKD, and ESRD) were made in accordance with the Kidney Disease: Improving Global Outcomes (KDIGO) guidelines [13,14,15]. Renal remission was defined as proteinuria <300 mg/g supcr with a GFR≥ 60 mL/min/1.73 m^2^ and using a glucocorticoid dose of ≤5 mg/day of prednisolone or equivalent. A relapse was defined as a >50% increase in proteinuria with hematuria or the reappearance of hematuria that was not explained by another medical condition other than vasculitis during a period after remission in patients with at least 3 months of follow-up. The definition of renal remission was determined by the investigators specifically for this study. Unfavorable outcomes were defined as established CKD (G3a-G5), renal replacement therapy, persistent proteinuria >500 mg/g supcr at the end of the follow-up, relapse disease, IgAVN-associated death, or a vasculitis damage index (VDI) score ≥1 at the end of the follow-up period.

### 2.3. Statistical Analysis

Statistical analyses were performed with the Statistical Package for the Social Sciences (SPSS) 26.0 (IBM, Chicago, IL, USA). The Kolmogorov–Smirnov test and the Shapiro–Wilk test were used to assess the normality of the data. Continuous variables were summarized as the mean ± standard deviation or median [interquartile range (IQR)] and categorical variables were summarized as percentages. Comparisons of continuous variables between groups were performed using a *t*-test or the Mann–Whitney U test after the distribution was evaluated, and the chi-square (χ^2^) test or Fisher’s exact test were used to compare categorical variables. The binary logistic regression analysis and multivariate Cox regression analysis methods were used to determine the predictors. A Kaplan–Meier estimation was performed to calculate the rate of the endpoint.

## 3. Results

### 3.1. Patient Characteristics and Presentation Signs

This study enrolled 49 adult patients (30 male; mean age: 49 ± 2.3 years) with newly diagnosed IgAVN. The median follow-up time was 22 (11–24) months. Of a total of 49 patients, 46 (94%) had skin involvement, 16 (32.6%) had joint involvement, and 11 (22.4%) had gastrointestinal involvement. Palpable purpura (94%) was the most common clinical sign at initial presentation, followed by hematuria (73.4%) and proteinuria (69.3%). Eleven patients (22.4%) had no renal pathology at initial presentation but developed renal involvement during follow-up. In patients who subsequently developed renal findings, the median time between the first visit and the renal findings was 30 (8–49) days. Regarding the renal findings, the patients most commonly had non-nephrotic proteinuria (73.4%) with active urinary sediment, 16.3% had nephrotic-range proteinuria, and 28.5% had acute kidney injury. Renal biopsy was performed in 55% of patients and a skin biopsy was performed in 94%. In all the patients with renal biopsies, pathological examination revealed increased mesangial cells and IgA deposits; all patients had evidence of mesangioproliferative glomerulonephritis. Glomerular crescent formation was present in 41% of the patients, but none of them had a crescent rate of more than 50%. The details of the histopathological findings can be found in the Supplementary Materials. The baseline demographic and clinical characteristics of the patients are shown in Table 1.

### 3.2. Immunosuppressive Management Within the Cohort

The drugs and drug doses used in the treatment were decided by the clinicians without any guidelines. All the patients received a glucocorticoid treatment (53.1% pulse methylprednisolone), and the median glucocorticoid dose was 500 mg/day of methylprednisolone. Azathioprine (AZA) was the most commonly preferred first glucocorticoid-tapering agent (41%), followed by cyclophosphamide (CYC) (31%) and mycophenolate mofetil (MMF) (6%). In seven patients (14%), the glucocorticoid-tapering agent was changed to another for various reasons. The drugs used in the treatment and their effects on the renal outcomes are shown in Table 2.

### 3.3. One-Year Renal Remission Rates and Factors Influencing Remission

A total of 36 patients reached the one-year follow-up point, and 86% of the patients were in remission at 12 months. The rate of NRP was significantly higher in patients who did not achieve renal remission at the end of the 12-month follow-up period (9.7% vs. 60%; *p* = 0.02). The one-year remission rates and the factors influencing remission are shown in Table 3.

### 3.4. Overall Unfavorable Outcomes and Associated Factors

A total of 10 patients had unfavorable outcomes at the end of the follow-up period. The presence of NRP was an independent risk factor for unfavorable outcomes [OR: 17.18; 95% CI: 1.31–224.95; *p =* 0.03]. The unfavorable outcomes and the factors associated with them are shown in Table 4. The vasculitis damage index (VDI) was used to assess disease- and medication-related damage. A total of 20% of the patients had a high VDI score (>1) at the end of the follow-up period. Patients with high VDI scores had a significantly higher total glucocorticoid exposure than those without (2.8 ± 1.3 gr vs. 4.4 ± 1.8 gr; *p* = 0.005) (Table 2). A total of 16% of the patients developed an infection that required hospitalization during the follow-up period. There was no difference in glucocorticoid exposure or the glucocorticoid-tapering agents used between patients with and without an infection that required hospitalization. However, the patients who developed an infection requiring hospitalization had a higher rate of IgAVN-associated AKI (62.5% vs. 22%; *p =* 0.02), and these patients were significantly older (mean 46 ± 15,3 vs. 65 ± 13,3; *p =* 0.002) (Table 5). The mortality rate was 4%. End-stage renal disease (ESRD) and renal replacement therapy occurred in only one patient, and this patient died due to sepsis in the 4th month. Another patient died of a heart attack at 32 months; he was in remission with no unfavorable outcomes. During the entire follow-up period, relapsed disease occurred in three (6%) patients (Table 1).

## 4. Discussion

It is generally accepted that renal involvement is more common and severe in adults with IgAV compared to children and is a major determinant of long-term morbidity and mortality [10]. The main management strategies of IgAVN are aimed at preventing the development of ESRD. In previous studies, the rate of ESRD in adult patients with IgAVN has been reported to be between 11% and 27% [16,17,18,19]. These ESRD rates seem to be comparable to those of ANCA-associated vasculitides (granulomatous polyangiitis and microscopic polyangiitis) and lupus nephritis [20,21]. In our study, only one (2%) patient developed ESRD. There may be several reasons for the lower rate of ESRD in this study. In previous studies, a significant proportion of patients (<40%) did not receive immunosuppressives other than glucocorticoids, whereas in this study, 87% of the patients received immunosuppressives in addition to the initial glucocorticoid treatment; the majority of previous studies had a retrospective design, whereas this study was prospective; patients were followed up closely at 3-month intervals and treatment changes were implemented promptly; and this study had a relatively shorter follow-up period (median of 22 months).

In cases of vasculitic renal involvement, it is crucial to define renal remission in clinical practice. However, there is not yet an internationally accepted definition of renal remission for IgAVN. Some previous studies have defined remission as the absence of proteinuria and hematuria with a stable GFR, while others have defined remission based on proteinuria alone. We believe that the dose of glucocorticoids used should be considered when defining renal remission, and we included the glucocorticoid dose in the definition of remission in this study. The remission rates were 86% at 12 months and 87% overall in this cohort. Previous studies have reported short-term remission rates of 10%-76.9% and long-term rates of 20%-80.8% [14,19,22,23]. Differences in the definition of remission, the lack of standardized treatment regimens, and genetic and geographical factors may be responsible for these different remission rates. It is clear that there is a need for internationally accepted, standardized remission definitions. Knowledge of the factors associated with remission rates and unfavorable renal outcomes may have implications for treatment and follow-up planning. In this study, NRP was a negative risk factor for achieving remission at 12 months. There is a paucity of studies that have examined the factors that influence long-term remission in IgAVN patients. However, a study published in 2019 by J Tan et al. revealed that IgAVN patients with nephrotic syndrome had lower remission and higher ESRD rates than others, which is similar to the findings of our study [24].

At the end of this study, 20% of the patients had unfavorable outcomes. In previous studies, an advanced age, the presence of hypertension, some histopathological findings (endocapillary proliferation, glomerular sclerosis and necrosis, and tubular atrophy and fibrosis), decreased GFR and proteinuria >1 g/day at presentation, and the presence of nephrotic syndrome were associated with unfavorable outcomes [18,24,25,26,27]. In our cohort, the rate of proteinuria >1 gr/day at presentation, the rate of NRP, and the five-factor score were significantly higher in the group of patients with unfavorable outcomes; in addition, the remission rates at 12 months were lower and the total glucocorticoid exposure was higher in this group. However, the multivariate regression analysis revealed only the presence of NRP as an independent risk factor for unfavorable outcomes. An elevated creatinine level and a low GFR at presentation have been highlighted as being associated with unfavorable outcomes in several previous studies [16,17,25]. In this study, there was no difference in unfavorable outcomes between patients with and without a low GFR at presentation; we believe this is related to the early and effective immunosuppressive treatment, given our high remission rates.

Looking at the treatment data in this study, all the patients received glucocorticoid therapy at different doses and for different durations, and 87% of the patients also received immunosuppressive therapy. In fact, it is still unclear how to treat adult patients with IgAVN. There are no high-level evidence-based studies on the efficacy of oral or intravenous pulse glucocorticoid therapy in adult patients with IgAVN. Data on the benefit of glucocorticoid therapy in IgAVN come from studies in children or patients with IgA nephropathy [28,29,30]. In 1998, Niaudet P and Habib R reported the clinical and histopathological benefits of pulse methylprednisolone in a study conducted with 38 pediatric patients with crescentic glomerulonephritis and nephrotic syndrome, although there was no control group in the study [30]. In our study, 53% of the patients received intravenous pulse methylprednisolone (methylprednisolone, >250 mg/day). There was no significant difference between the pulse methylprednisolone and non-pulse groups in terms of the 12-month remission rates and the overall unfavorable outcomes. In this study, AZA was the most frequently preferred drug as the first-line glucocorticoid-sparing agent, followed by CYC and MMF. There are no studies revealing that AZA is effective in adult patients with IgAVN, although it is a frequently preferred drug for these patients. Limited data on the efficacy of AZA come from studies with a low level of evidence in pediatric patients [31,32]. Similarly, although the literature data on MMF are limited, two retrospective studies conducted in China have previously reported that MMF may be beneficial in adult patients with IgAVN [23,33]. In our cohort, 33% of the patients received MMF as the glucocorticoid-sparing agent. Although CYC is now a relatively less preferred drug in rheumatic diseases than in the past due to the risk of toxicity, it is recommended as a first-line drug because of its potency and rapid effect in severe life- or organ-threatening diseases (e.g., ANCA-associated vasculitis and lupus nephritis) [34,35]. The efficacy of CYC in adult patients with IGAVN has not been demonstrated in the limited number of previous studies [22,36]. However, the current KDIGO guidelines recommend the use of CYC in combination with steroids in the presence of rapidly progressive glomerulonephritis *(RPGN)* in patients with IgAVN [14]. In our cohort, 44% of the patients receiving CYC presented with AKI, 73% had crescent formation (<50%) on renal biopsies, the mean proteinuria level was 2.3 g/day, 25% had an unfavorable outcome, and 13% developed stage G3a CKD. These data suggest that clinicians may prefer CYC in cases with severe disease at presentation. There was no significant difference in the remission rates or unfavorable outcomes between the selected first-line glucocorticoid-sparing agent groups. Frankly, there are no specific guidelines for the treatment of adult IgAVN. In the current KDIGO guideline, IgAVN is included with IgAN to a limited extent. It is emphasized that these recommendations apply only to renal involvement and are largely based on data from studies of IgAN. However, the guideline recommends strict blood pressure control and lifestyle modification for at least 3 months with ACEis/ARBs (angiotensin-converting enzyme inhibitors/angiotensin receptor blockers) prior to steroid treatment for IgAN without RPGN, and the same recommendation is made for IgAVN. Despite the similarity in the histopathological findings, the clinical courses of these two diseases are very different. Even in the absence of RPGN, this recommendation worries rheumatologists. It is clear that more disease-specific studies are needed to formulate recommendations with high-level evidence for the treatment of IgAVN [14].

In systemic vasculitis, it is very important for clinicians to objectively determine disease- and treatment-related damage. There are no studies in the literature that have investigated disease/treatment-related damage in adult patients with IgAVN. However, Gazel U et al. published a study on the assessment of damage in patients with IgAV in 2020. In that study, the VDI score was used to perform a damage assessment; in total, 24.7% of the patients had at least one damage item, and the damage was related to the disease itself rather than the treatment [37]. In this study, the VDI was also used to perform a disease/treatment-related damage assessment. A total of 22% of the patients had at least one damage item. The total glucocorticoid exposure was significantly higher in the high-damage score group than in the no-damage group. However, high damage scores were more related to the disease than to the treatment (64% vs. 36%).

In previous reports of adult IgAVN cohorts, serious infection rates have been reported to be between 2% and 4% [16,22,23]. In this study, during the follow-up period, 16% of our patients developed infections that required hospitalization. There was no difference between patients with and without serious infections in terms of the total glucocorticoid exposure and the glucocorticoid-tapering agents used. However, patients who developed serious infections were significantly older than those who did not, and the rate of AKI at presentation was significantly higher in this group. It is known that AKI creates an immunosuppressive status, and infections are important causes of mortality during the recovery period in patients with an AKI [38,39]. In a study published in 2016, Hong S et al. showed that the mortality was higher in patients with late-onset IgAV compared with early-onset patients and that infections were an important cause of mortality [40]. The higher rate of serious infections in this study compared to previous studies may be explained by the more frequent use of immunosuppressives and the more effective doses. Literature data on this issue are very limited and more detailed studies are needed. In previous studies, the relapse rate in patients with IgAVN has been reported to vary between 13% and 22.6% [23,33,40]. In this study, the relapse rate was 6%. Similarly to remission, a standardized definition for relapse has not been established to date. Differences in the definition of relapse and the treatments administered may explain the variable relapse rates. In our cohort, one patient died from sepsis and another patient died from a heart attack. The patient who died from sepsis was not in remission and was receiving renal replacement therapy. In the cohort analysis published by Pillebout E et al. in 2002, the mortality rate was 26% after 14.8 years of follow-up. This was a high rate, and the most common cause of mortality was cancer [17]. Subsequently, Coppo R et al. reported a mortality rate of 5.1% in a cohort with a follow-up of 5.5 years in 2006; Oh HJ et al. reported a rate of 2.2% in a cohort with a follow-up of 5.2 years in 2012; and Guan Y et al. reported a rate of 7.4% in a cohort with a follow-up of 8.5 years in 2023 [19,41,42]. The most common causes of death were cancer/cerebrovascular attack, unknown causes, and infections, respectively. It was striking that cancer was the leading cause of mortality and that disease-related complications were in the background. It is clear that more comprehensive studies are needed to determine the factors associated with mortality in patients with IgAVN.

Our study has some limitations. Only 55% of the patients included in the study had a renal biopsy, which may have influenced the clinicians’ treatment preferences or the histopathological findings that affected the prognosis. The cohort consisted of a relatively small number of patients, resulting in wide confidence intervals for the parameters found to be significant in the regression analyses. Finally, there was no control group for the treatment groups.

## 5. Conclusions

These results suggest that contrary to popular belief, the renal prognosis of adults with IgVAN is not poor if they receive effective immunosuppressive therapy. The presence of NRP is associated with low remission rates, and it has predictive value for unfavorable outcomes. Patients with NRP should be followed-up more closely and treated more stringently. Effective immunosuppressive treatment and close follow-up can achieve high remission rates and significantly reduce the risk of ESRD in adults with IgAVN, but immunosuppressive therapy may also carry a high risk of infection, particularly in the elderly. It is clear that further prospective controlled trials are needed for the rational management of adult patients with IgAVN. Future research should focus on the treatment of different subgroups (for the elderly, in cases of nephrotic disease, etc.).

## Figures and Tables

**Table 1:** Demographics and clinical characteristics of the patients.

	*N* = 49
Age, year; mean ± SD	49 ± 2.3
Male; *n*/*N* (%)	30 (6.2)
Follow-up time, month; median (IQR)	22 (11–24)
Organ system involvement; *n*/*N* (%)	
Skin	46 (94)
Joint	16 (32.6)
Gastrointestinal	11 (22.4)
Renal	49 (100)
Overall renal involvement; *n*/*N* (%)	
Nephrotic-range proteinuria	8 (16.3)
Non-nephrotic proteinuria	36 (73.4)
Isolated hematuria	5 (10.2)
Acute kidney injury	14 (28.5)
Overall renal outcomes; *n*/*N* (%)	
Persistent proteinuria (>500 mg/24 h)	6 (12)
Relapse	3 (6)
Chronic kidney disease (G3a–G5)	4 (8)
G3a	1 (2)
G3b	1 (2)
G4	1 (2)
G5	1 (2)
Established renal replacement therapy	1 (2)
VDI score (≥1); *n*/*N* (%)	10 (20)
Mortality; *n*/*N* (%)	
Sepsis	1 (2)
Myocardial infarction	1 (2)

SD: standard deviation; VDI: vasculitis damage index; G3a-G5: Kidney Disease: Improving Global Outcomes (KDIGO) chronic kidney disease stages—G3a: GFR 45–59; G3b: GFR 30–44; G4: GFR15–29; G5: GFR < 15—IQR: interquartile range.

**Table 2:** Treatment data of the patients.

	All of the Patients *N* = 49	Remission at 12 Months (+) vs. (−)/*p*-Value	VDI Score (≤1) vs. (>1)/*p*-Value	Unfavorable Renal Outcomes (+) vs. (−)/*p*-Value
All of the steroid-sparing agents; *n*/*N* (%)				
CYC	16 (33)			
AZA	25 (51)			
MMF	16 (33)			
MFA	1 (2)			
COLCH	5 (10)			
LEF	1 (2)			
TOCI	1 (2)			
First steroid-sparing agent; *n*/*N* (%)				
CYC	15 (31)	22.6 vs. 20/0.691	23.1 vs. 30/0.693	40 vs. 28.2/0.478
AZA	20 (41)	41.9 vs. 60/0.632	41 vs. 50/0.61	40 vs. 41/0.993
MMF	3 (6)			
COLC	3 (7)			
LEF	1 (2)			
None	7 (14)			
Steroid-sparing agent after cyclophosphamide; *n/N* (%)				
AZA	5/13 (38)	40 vs. 0/0.633	27.3 vs. 50/0.569	50 vs. 27.3/0.409
MMF	8/13 (62)	60 vs. 100/0.634	54.5 vs. 50/0.66	50 vs. 54.5/1.000
Steroid-sparing agent switch; *n/N* (%)				
AZA > MMF (adverse event)	4/42 (10)			
AZA > MMF (inadequate response)	1 (2)			
CYC > MMF (adverse event)	1 (2)			
CYC > AZA > TOCI (inadequate response for arthritis)	1 (2)			
Initial glucocorticoid dosage (prednisolone or equivalent MP); *n/N* (%)				
Pulse (≥250 mg/day)	26 (53.1)	48.4 vs. 80/0.342	48.7 vs. 70/0.296	70 vs. 48.7/0.22
1 mg/kg/day	8 (16.3)	19.4 vs. 0/0.64	17.9 vs. 10/0.477	10 vs. 17.9/0.541
0.5–1 mg/kg/day	7 (14.3)	9.7 vs. 0/0.633	12.8 vs. 20/0.624	20 vs. 12.8/0.565
≤0.5 mg/kg/day	8 (16.3)	19.4 vs. 0/0.561	15.4 vs. 0/0.32	0 vs. 15.4/0.187
Total glucocorticoid (prednisolone) exposure (gr); mean ± SD	3.1 ± 0.2	2.2 ± 1.3 vs. 3.5 ± 1.2/***0.01***	3.0 ± 1.6 vs. 4.6 ± 1.6 /0.06	4.3 ± 1.8 vs. 2.8 ± 1.3 /***0.006***

CYC: cyclophosphamide; AZA: azathioprine; MMF: mycophenolate mofetil; MFA: mycophenolic acid; COLC: colchicine; LEF: leflunomide; TOCI: tocilizumab; MP: methylprednisolone; VDI: vasculitis damage index; SD: standard deviation.

**Table 3:** Factors associated with long-term renal remission.

	Renal Remission (+) at 12 Months *N* = 31	Renal Remission (−) at 12 Months *N* = 5	*p*-Value
Age; mean ± SD	48.6 ± 16	56.6 ± 19.8	0.322
Male; *n*/*N* (%)	17 (54.8)	2 (40)	0.651
Renal involvement; *n*/*N* (%)			
Nephrotic-range proteinuria	3 (9.7)	3(60)	<***0.02***
Non-nephrotic proteinuria	22 (71)	2 (40)	0.333
Isolated hematuria	4 (12.9)	0 (0)	0.398
Isolated proteinuria	2 (6.5)	0 (0)	0.559
Acute kidney injury	10 (32.3)	2 (40)	0.733
Other system involvement; *n*/*N* (%)			
Skin involvement	31 (100)	4 (80)	0.135
Joint involvement	10 (32.3)	1 (20)	0.515
Gastrointestinal involvement	7 (22.6)	0 (0)	0.554
Baseline laboratory findings; *n*/*N* (%)			
CRP (mg/L); median (IQR)	40.8 (7–116)	13.5 (2.1–22.8)	0.112
GFR; median (IQR)	102 (80–119)	93 (45–119)	0.742
Serum albumin, <3.5 gr/dL; *n*/*N* (%)	7 (22.6)	0 (0)	0.556
Serum IgA level; mean ± SD	3.6 ± 1.3	2.9 ± 1	0.513
SII; median (IQR)	975 (516–1585)	807 (473–1199)	0.478
BVAS; median (IQR)	12 (7–13)	12 (7–13.5)	0.732
Five-factor score, >1; *n*/*N* (%)	14 (45.2)	3 (60)	0.651
Initial glucocorticoid dosage (prednisolone), mg/day; median (IQR)	80 (60–1000)	1000 (500–1000)	0.09
Comorbid systemic disease; *n*/*N* (%)			
Hypertension	10 (32.1)	2 (40)	0.541
Type 2 diabetes mellitus	10 (32.2)	1 (20)	0.512
Comorbid rheumatic disease; *n*/*N* (%)			
FMF	3 (9.7)	0 (0)	0.631
AS	2 (6.5)	0 (0)	0.739
Predisposing factor; *n*/*N* (%)			
None	17 (54.8)	3 (40)	0.615
Infection	11 (35.5)	3 (60)	0.351
Drug	7 (22.6	0 (0)	0.312

CRP: C-reactive protein; IQR: interquartile range; GFR: glomerular filtration rate; SII: systemic immune–inflammation index; BVAS: Birmingham vasculitis activity score; FMF: familial Mediterranean fever; AS: ankylosing spondylitis; SD: standard deviation.

**Table 4:** Factors associated with unfavorable outcomes and multivariate regression analysis.

	Unfavorable Outcomes (+) *N* = 10	Unfavorable Outcomes (−) *N* = 39	*p*-Value	Regression Analysis	
				OR (95% CI)	*p*-Value
Age, >60 years; *n*/*N* (%)	6 (60)	8 (20.5)	***0.01***	1.32 (0.02–73.32)	0.891
Male; *n*/*N* (%)	5 (50)	25 (64.1)	0.418		
Baseline laboratory					
CRP (mg/L); median (IQR)	22.8 (9.7–46.8)	40.8 (6.5–121)	0.444		
ESR (mm/h); mean ± SD	41.1 ± 30.7	43.3 ± 27.7	0.846		
GFR; median (IQR)	95 (70–115)	93 (80–110)	0.92		
Serum albumin (gr/dL); mean ± SD	3.9 ± 0.5	3.8 ± 0.6	0.591		
BVAS; median (IQR)	10 (6–13.2)	12 (9–15)	0.215		
Five-factor score, >2; *n/N* (%)	4 (40)	3 (7.7)	***0.02***	0.60 (0.23–15.97)	0.764
SII; median (IQR)	689 (335–1110)	978 (516–2135)	0.483		
Baseline renal condition; *n/N* (%)					
Crescent on biopsy (+)	4/4 (100)	10/16 (62.5)	0.266		
Proteinuria (>500 mg/day)	9 (90)	19 (48.7)	***0.03***	3.52 (0.33–37.14)	0.291
Acute kidney injury	2 (20)	4 (10.3)	0.581		
Nephrotic-range proteinuria (>3.5 gr/day)	6 (60)	2 (5.1)	***0.001***	***17.18 (1.31–224.95)***	***0.03***
Other system involvement; *n/N* (%)					
Skin	9 (90)	37 (94.9)	0.56		
Joint	3 (30)	13 (33)	0.84		
Gastrointestinal	2 (20)	9 (23.1)	0.83		
Renal remission; *n/N* (%)					
at 12 months	4/8 (50)	27/28 (96.4)	***0.005***	0.06 (0.02–2.53)	0.14
Glucocorticoid dosage,					
Pulse steroid (>250 mg/day/MP); *n/N* (%)	7 (70)	19 (48.7)	0.22		
Total steroid exposure (gr/PR); mean ± SD	4.3 ± 1.8	2.8 ± 1.3	***0.006***	2.49 (0.93–6.60)	0.06
First steroid-tapering agent; *n/N* (%)					
AZA	4 (40)	16 (41)	0.95		
CYC	4 (40)	11 (28.2)	0.47		
Comorbid systemic disease; *n/N* (%)					
Hypertension	4 (40)	11 (28.2)	0.47		
Type 2 diabetes mellitus	4 (40)	11 (28.2)	0.47		
Comorbid rheumatic disease; *n/N* (%)					
FMF	0 (0)	5 (12.8)	0.23		
AS	0 (0)	2 (5.1)	0.46		
Predisposing factor; *n/N* (%)					
None	2 (2)	18 (46.2)	0.16		
Infection	6 (60)	13 (33.3)	0.12		
Drug	1 (10)	11 (28.2)	0.23		

CRP: C-reactive protein; IQR: interquartile range; ESR: erythrocyte sedimentation rate; GFR: glomerular filtration rate; SII: systemic immune–inflammation index; BVAS: Birmingham vasculitis activity score; FMF: familial Mediterranean fever; AS: ankylosing spondylitis; AZA: azathioprine; CYC: cyclophosphamide; MP: methylprednisolone; PR: prednisolone; SD: standard deviation.

**Table 5:** Factors associated with infections that required hospitalization.

	Serious Infection (+) *N* = 8	Serious Infection (−) *N* = 41	*p*-Value
Age; mean ± SD	46 ± 15.3	65 ± 13.3	***0.002***
Gender; *n*/*N* (%)			
Male	5 (62)	25 (61)	0.931
Female	3 (38)	16 (39)	0.942
Comorbidities; *n*/*N* (%)			
Type 2 DM	4 (50)	11 (23)	0.072
FMF	1 (12.5)	4 (9.8)	0.601
AS	0 (0)	2 (4.9)	0.694
Total steroid exposure (gr); mean ± SD	3.6 ± 1.2	3.1 ± 1.7	0.382
First steroid-tapering agent used; *n*/*N* (%)			
CYC	3 (37)	12 (29)	0.68
AZA	2 (25)	18 (43.9)	0.444
MMF	2 (25)	1 (2.4)	0.062
Renal conditions during the follow-up period; *n*/*N* (%)			
NRP	3 (37.5)	5 (12.2)	0.113
AKI	5 (62.5)	9 (22)	***0.02***
CKD	2 (25)	2 (4.9)	0.125

SD: standard deviation; FMF: familial Mediterranean fever; AS: ankylosing spondylitis; CYC: cyclophosphamide; MMF: mycophenolate mofetil; NRP: nephrotic-range proteinuria; AKI: acute kidney injury; DM: diabetes mellitus; AZA: azathioprine; CKD: chronic kidney disease.

## Data Availability

The data can be provided by the authors upon request, with the removal of identifiable information and the anonymization of dates.

## Supplementary Materials

The following supporting information can be downloaded at: https://www.mdpi.com/article/10.3390/diagnostics15080957/s1, Table S1: Histopathological features of the patients. Dataset S1: Dataset form.
