# Supplementary material for: Disease Course and Long-Term Outcomes in Adult IgA Vasculitis Nephritis: A Prospective Observational Study
Source: Diagnostics (Basel). 2025 Apr 10;15(8):957. doi: 10.3390/diagnostics15080957 (PMC12025811; doi:10.3390/diagnostics15080957)
Supplement: Supplementary file 1 [file diagnostics-15-00957-s001.zip › Supplementary File_ Histopathology_Table.pdf]

Supplementary Material

Table S1. Histopathological features of the patients

	N=27
DIF findings, n/N (%)	
IgA	27 (100)
IgG	3 (11.1)
IgM	6 (22.2)
Fibrinojen	8 (36.3)
C3	18 (66.6)
C1q	0 (0)
Kappa	20 (74)
Lambda	18 (66.6)
MEST-C score parameters, n/N (%)	
M0, M1	10 (37), 17 (63)
E0, E1,	16 (59.2), 11 (40.8)
S0, S1	23 (85.1), 4 (14.9)
T0, T1, T2	21 (77.7), 6 (22.3), 0 (0)
C0, C1, C2	16 (59), 11 (41)

DIF: direct immunofluorescence, MEST-C/ M: Mesangial hypercellularity, E: Endocapillary hypercellularity, S: Segmental glomerulosclerosis, T: Tubular atrophy/interstitial fibrosis, C: Cellular/fibrocellular crescents
